# Oxidative Stress and Inflammation in COVID-19-Associated Sepsis: The Potential Role of Anti-Oxidant Therapy in Avoiding Disease Progression

**DOI:** 10.3390/antiox9100936

**Published:** 2020-09-29

**Authors:** Jesús Beltrán-García, Rebeca Osca-Verdegal, Federico V. Pallardó, José Ferreres, María Rodríguez, Sandra Mulet, Fabian Sanchis-Gomar, Nieves Carbonell, José Luis García-Giménez

**Affiliations:** 1 Center for Biomedical Network Research on Rare Diseases (CIBERER), Institute of Health Carlos III, 46010 Valencia, Spain; jesus.beltran@ext.uv.es (J.B.-G.); federico.v.pallardo@uv.es (F.V.P.); 2 Department of Physiology, Faculty of Medicine & Dentistry, University of Valencia, 46010 Valencia, Spain; rebeca.osca@gmail.com (R.O.-V.); fabian.sanchis@uv.es (F.S.-G.); 3 INCLIVA Biomedical Research Institute, 46010 Valencia, Spain; ferreresj@gmail.com (J.F.); mariarodriguezgimillo@gmail.com (M.R.); sandramuletmascarell@gmail.com (S.M.); 4 EpiDisease S.L. (Spin-Off CIBER-ISCIII), Parc Científic de la Universitat de València, 46980 Paterna, Valencia, Spain; 5 Intensive Care Unit, Clinical University Hospital of Valencia, 46010 Valencia, Spain

**Keywords:** SARS-CoV-2, sepsis, ACE2, pyroptosis, NETosis, cytokine storm

## Abstract

Since the severe acute respiratory syndrome coronavirus 2 (SARS-CoV-2) outbreak emerged, countless efforts are being made worldwide to understand the molecular mechanisms underlying the coronavirus disease 2019 (COVID-19) in an attempt to identify the specific clinical characteristics of critically ill COVID-19 patients involved in its pathogenesis and provide therapeutic alternatives to minimize COVID-19 severity. Recently, COVID-19 has been closely related to sepsis, which suggests that most deceases in intensive care units (ICU) may be a direct consequence of SARS-CoV-2 infection-induced sepsis. Understanding oxidative stress and the molecular inflammation mechanisms contributing to COVID-19 progression to severe phenotypes such as sepsis is a current clinical need in the effort to improve therapies in SARS-CoV-2 infected patients. This article aims to review the molecular pathogenesis of SARS-CoV-2 and its relationship with oxidative stress and inflammation, which can contribute to sepsis progression. We also provide an overview of potential antioxidant therapies and active clinical trials that might prevent disease progression or reduce its severity.

## 1. Introduction

Coronavirus disease 2019 (COVID-19) is spreading rapidly all over the world, affecting millions of people [1]. In fact, it was declared a pandemic by the World Health Organization (WHO) on 11 March of the present year [2] and fears have increased with a more widespread “second wave”.

Clinically, the majority of patients infected by the severe acute respiratory syndrome coronavirus 2 (SARS-CoV-2) are minimally symptomatic or asymptomatic, but ~5% of patients show severe lung injury and/or multiple organ dysfunction and become septic shock. Thus, mortality ranges between 7–15%, depending on the hospital and country [3]. Also, critically ill COVID-19 patients often develop typical clinical manifestations of shock [4], including cold extremities and weak peripheral pulses [5], metabolic acidosis, and abnormal coagulation parameters (e.g., D-dimer, abnormal fibrin degradation products levels) [6], which is indicative of microcirculation dysfunction [5,7]. COVID-19 is characterized by localized and systemic coagulopathies [8] and disseminated intravascular coagulation (DIC) [9], named “COVID-associated coagulopathy,” which is directly associated with poor outcomes. Other metabolism-related alterations found in COVID-19 patients are a consequence of liver and kidney dysfunction and severe lung injury [5]. Numerous patients with severe COVID-19 meet the Third International Consensus Definitions for Sepsis (Sepsis-3) [4,10], which defines sepsis as “a life-threatening condition that arises when the body’s response to infection damages the host’s own tissues” [10]. Overall, most deaths in critically ill COVID-19 patients are caused by sepsis [11,12], as we also recently reported [4].

Over the past months, the COVID-19 crisis has seriously jeopardized the capabilities of most healthcare systems worldwide. Intensive care unit (ICUs) capacity and resource limitations have been identified as a major bottleneck for proper assistance of COVID-19 patients [13], increasing the risk of adverse outcomes. Therefore, in this context, the validation of new therapeutic strategies and treatments are of special relevance to reduce disease progression to severe phenotypes.

## 2. Molecular Pathogenesis of COVID-19

Understanding the relationship of molecular mechanisms dysregulated or mediated by SARS-CoV-2 which can lead to sepsis, and learning how inflammation, metabolic alterations, and oxidative stress are produced, are current clinical needs that are essential to improving the COVID-19 prognosis and providing a rational treatment strategy. In this section we describe relevant molecular mechanisms underlying COVID-19 physiopathology, including oxidative stress and immune response dysregulation, and their interconnection.

### 2.1. Oxidative Stress

**Figure 1:**
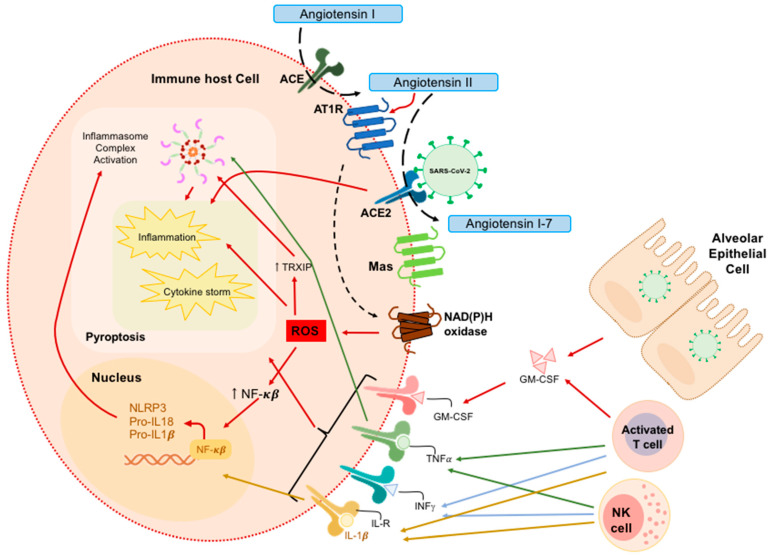
Molecular pathogenesis of SARS-CoV-2. SARS-CoV-2 virus can bind to specific receptors in host cells, such as alveolar epithelial cells or immune cells, by mediating an inflammatory cascade through inflammasome activation, usually through NLRP3, and by damaging, or even killing, host cells. Immune host cells, like macrophages and monocytes, are activated by the virus on a direct or indirect pathway, and contribute to the host response by causing inflammation and a cytokine storm. Other immune cells, such as NK cells and T-cells, can contribute to the immune response. On the other hand, Ang II is processed by ACE2 into Ang I-7. Ang II contributes to ROS production in an NAD(P)H-dependent mechanism thanks to the ACE2–Ang-(1–7)–Mas axis. AT1R mediates the production of ROS through a mechanism dependent on NADH and NADPH oxidases. ROS contributes to nuclear factor-kB (NF-κB) and TXNIP overexpression. NF-κB increases the expression of NLRP3, pro-IL-18, and pro-IL-1β whilst TXNIP modulates the structure of NLRP3, thereby allowing NLRP3 inflammasome assembly and facilitating pro-caspase-1 (pro-casp-1) autocleavage. Arrows denote activation. ACE2: angiotensin-converting enzyme 2; GM-CSF: Granulocyte-macrophage colony-stimulating factor; TNF: Tumor Necrosis Factor; INFγ: Interferon gamma; IL-R: Interleukin Receptor; AT1R: Angiotensin type 1; NADPH oxidase: nicotinamide adenine dinucleotide phosphate oxidase; ROS: reactive oxygen species. TXNIP: thioredoxin interacting/inhibiting protein. This figure is adapted from the work of Merad et al. [33].

Oxidative stress has recently been proposed as a key player in COVID-19 [14,15]. Importantly, Lei et al. have recently demonstrated that the affinity of SARS-CoV-2 to ACE2 is 1–2-fold higher than the previous virus SARS-CoV [16]. The mechanism involves the activity of ACE2 cleaving the octapeptide angiotensin II (Ang II), which is previously generated by ACE. Since Ang II is a powerful vasoconstrictor with a key role in the elevation of blood pressure, its processing by ACE2 induces vasodilation, accentuated by the generation of Ang 1-7, a peptide with potent vasodilator roles which is generated during this process. The binding of SARS-CoV-2 to ACE2 causes the virus to enter cells and in turn reduces the bioavailability of ACE2. Due to the protective function of ACE2, the reduction of its levels is related to adverse clinical phenotypes, and its key role in the pathogenesis of SARS-Cov-2 has been described [17]. Evidence has shown that Ang II regulates nicotinamide adenine dinucleotide phosphate (NADPH) oxidase (NOX) activation [18,19,20,21], when Ang II binds to angiotensin type 1 (AT1R) [22]. NOX activation is one of the major contributors to the formation of ROS (including superoxide radical anion (O_2_^•−^) and hydrogen peroxide (H_2_O_2_)). Therefore, the reduction in ACE2 bioavailability after SARS-CoV-2 binding allows Ang II to be available to interact with AT1R, which mediates signals to activate NADPH oxidase and induce oxidative stress and inflammatory responses (Figure 1), which in turn contribute to the severity of COVID-19 [23,24].

Violi et al. have shown that NADPH oxidase-2 (NOX-2) is overexpressed in hospitalized COVID-19 patients, causing increased oxidative stress [25]. In agreement with these results, other authors have shown in macrophages that NOX-2 blockade improves the disease phenotypes thanks to a decrease in oxidative stress [26]. Interestingly, it is noteworthy that when stimulated by proinflammatory cytokines and other agonists, endothelial cells can mobilize NOX proteins [27], contributing to local oxidative stress, which in turn produces endothelial dysfunction [28].

Other relevant mechanisms related with ACE2 and oxidative stress in the pathogenesis of COVID-19 include the endothelial dysfunction produced as consequence of ROS produced by NAD(P)H oxidase, which reduces the bioavailability of nitric oxide, which in turn results in vasoconstriction, inflammation, redox imbalance, and endothelial dysfunction. Thus, when ACE2 is dysfunctional or its levels are reduced as a consequence of the SARS-CoV-2, the classic renin-angiotensin–aldosterone system (RAAS), particularly the ACE2–Ang-(1–7)–Mas axis, becomes a potent pro-oxidant system in vessels [29].

Further important mechanisms which may occur in COVID-19 include the release of iron into the blood stream from red blood cells, which can mediate Fenton and Haber–Weiss reactions to produce oxidative stress. This mechanism occurs because SARS-CoV-2 attacks hemoglobin (Hb) groups in the red blood cells, thereby producing the release of free Fe(III) ions from the heme groups to the bloodstream [30], which in turn produces an increase in ferritin levels [31]. Hemoglobinopathy and iron dysmetabolism, mediated by the virus, may contribute to clinical syndromes highlighted during COVID-19 including oxidative stress, ferroptosis, lipid peroxidation, and mitochondrial damage, among others [32].

### 2.2. Inflammation and Immune Response

Coronaviruses subtypes like SARS-CoV, and specially SARS-CoV-2, are able to actively induce the so-called ‘cytokine storm’ by mediating an exacerbate production and release of proinflammatory cytokines [34], which confirms the high levels of inflammatory markers found in COVID-19 patients [35,36,37,38,39]. Interestingly, one of the markers found is the nonspecific C-reactive protein, a widely used biomarker for sepsis diagnosis. Furthermore, elevated levels of inflammatory cytokines and chemokines have been associated with COVID-19 severity and death [35,36,37,38,39]. Increased plasma concentrations of interleukin (IL), such as IL-1β, IL-2, IL-6, IL-7, IL-8, IL-10, or IL-17, interferon (IFN)γ, IFNγ-inducible protein 10, monocyte chemoattractant protein 1 (MCP1), granulocyte-macrophage colony-stimulating factor (GM-CSF), macrophage inflammatory protein 1α, and tumor necrosis factor-alpha (TNFα), among others, have been found as inflammatory mediators in COVID-19 [39,40,41,42,43,44]. Importantly, macrophages and neutrophils also play a potential pathological role during SARS-CoV-2 infection [33] by producing numerous ROS, including but not limited to H_2_O_2_, O_2_^•−^ and hydroxyl radical (^•^OH) [45]. Oxidative stress affects the immune system throughout altering immune cell function and inflammatory response [46,47].

Systemic cytokine profiles observed in severe COVID-19 patients present similarities to those observed in cytokine release syndromes, such as sepsis, with increased production of cytokines such as IL-6, IL-8, TNFα and other pro-inflammatory chemokines, including CC chemokine ligand 2 (CCL2), CCL3 and chemokine ligand 10 CXC (CXCL10). Another key inflammation mechanism, pyroptosis, also seems to be activated in both COVID-19 and sepsis [48,49,50]. This led to hypothesize that the dysregulated activation of a wide range of hyperinflammatory factors associated with COVID-19 could cause sepsis and septic shock in these patients [4,5].

### 2.3. Inflammasome as a Contributor to Inflammation

It has been previously found in SARS-CoV infected patients that inflammation appears to be preceded by the activation of pyroptosis. In these patients, the virus mediated the release of IL-1β during early infection [48,49,50]. Importantly, the rapid progression to severe phenotypes in COVID-19 patients coincides with an abrupt shift from the NLRP3 cytokine storm to a compensatory immunosuppressive state [44].

Some authors postulate that the NOD-like receptor family pyrin domain containing 3 (NLRP3), the major protein component of the inflammasome, may play a central role in the predisposition to the cytokine storm observed in certain COVID-19 patients [51] (Figure 1). Interestingly, oxidative stress is an inductor of the NLRP3 inflammasome by contributing to NLRP3 inflammasome formation and activation [52,53,54]. The mechanism of induction of NLRP3 inflammasome by ROS is mediated through nuclear factor-kB (NF-κB) and thioredoxin interacting/inhibiting protein (TXNIP). Specifically, the deubiquitination of TXNIP has been suggested to modulate NLRP3 oligomerization [54,55,56]. Oxidative stress also activates NF-κB which in turn, after its nuclear translocation, activates the expression of NLRP3, pro-IL-18 and pro-IL-1β, increasing the activation of NLRP3 inflammasome and subsequent pro-caspase-1 (pro-casp-1) autocleavage [57,58,59] (Figure 1).

Finally, some authors have postulated oxidative stress as an important player in the activation of the inflammasome during SARS-CoV-2 infection [60] and have proposed proteins such as TRPM2 as key factors that linking oxidative stress to NLRP3 inflammasome activation [61].

Mechanistically, pyroptosis is an inflammatory molecular pathway mediated by multiprotein complexes termed inflammasomes that triggers a high inflammatory response and cell death [62]. Inflammasomes assemble around sensor proteins, such as NLRP3, which are activated when a wide range of molecules, such as viral particles, are recognized as pathogen-associated molecular patterns (PAMPS) and/or damage-associated molecular patterns (DAMPS) [63] (Figure 1).

Notably, viruses have developed different strategies to modulate inflammasome activation, evade host detection and increase the spread of infection [64,65]. However, pro-inflammatory cytokines not only mobilize the host immune defense, but also drive pathogenic inflammation [64]. During viral infection, inflammation can play anti-viral and pro-viral roles. Inflammasomes form part of the innate anti-viral response by restricting viral infection and replication [66]. SARS-CoV-2 activates the inflammasome probably by binding ACE2, which may, in turn, lead to inflammatory cascade activation, and ultimately to increased inflammation [67] (Figure 1). The inflammation caused by the virus induces antiviral responses, but unfortunately, it could also facilitate their viral dissemination through the release of many virions into the bloodstream, which would allow to spread to other parts of the body [66]. Nevertheless, whether inflammasome plays a positive or negative role in COVID-19 is still unknown.

Although the knowledge on this inflammasome molecular process is continuously increasing, the temporal contribution of inflammasome-induced IL-1β to viral clearance and/or COVID-19 severity remains to be investigated [33].

### 2.4. NETosis as an Innate Immune Mechanism Contributing to Inflammation

**Figure 2:**
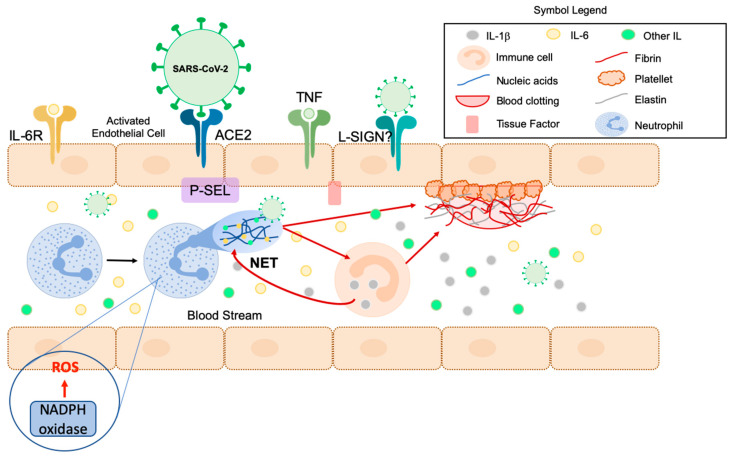
NETosis activated by SARS-CoV-2. SARS-CoV-2 and cytokines activate endothelial cells, which are able to produce adhesion molecules (e.g., P-SEL) and recruit neutrophils, which release NETs composed of nucleic acids, histones, and other proteins. NETs can bind to the SARS-CoV-2 present in the bloodstream and neutralize it. NETs can induce different immune cells to secrete IL-1β, which enhances NET formation in several diseases by providing positive feedback and accelerating thrombus formation. In addition, NADPH oxidase can produce ROS which activates NETosis. At the same time, the IL-6 receptor binds to IL-6, which enters the bloodstream and interacts with NETs. IL-1β is able to induce IL-6 expression which, in turn, is able to induce fibrinogen expression by increasing fibrin release. The virus’ endothelial damage can expose a tissue factor in activated endothelial cells capable of stimulating the coagulation pathway, which consists in fibrin deposition and blood clotting. At the same time, NETs also activate the coagulation contact pathway and bind activated platelets to contribute to amplify blood clotting. ACE2: angiotensin-converting enzyme 2; TNF: tumor necrosis factor; NET: neutrophil extracellular trap; IL-6R: Interleukin 6 receptor; P-SEL: P-Selectin. This figure is adapted from the work of Merad et al. [33].

Among other molecular mechanisms that have been proposed as playing a central role in COVID-19 pathogenesis related to sepsis, NETosis has appeared as a key relevant mechanism [34] (Figure 2). NETosis is an innate immune mechanism that protects human body from pathogens. Neutrophils release granular proteins and chromatin to form an extracellular fibrillar network matrix known as NET (neutrophil extracellular trap) via an active process, by which NET can bind to pathogens (e.g., bacteria, fungi, viruses) and neutralize them (Figure 2). Although NETs play a crucial role in host defense against pathogens, collateral damage from sustained NET formation also stimulates many complications, including those that occur during viral infections [68]. NETs release involves ROS formation by NOX proteins [69], which act intracellularly to initiate the formation of NETs [70].

The SARS-CoV-2 infection has been also associated with NET formation. Rises in circulating NETs levels have been previously found in patients with ARDS [71]. Likewise, neutrophils appear “primed” to activate the NETosis process in patients with pneumonia-associated ARDS, which may explain the correlation between this priming and the blood NET levels with severity and mortality [72,73,74,75,76]. Furthermore, oxidative stress may increase the formation of NETs and alter the adaptive immune system by contributing to T-cell suppression [77]. NETs can also induce the production of certain immune cells, such as monocytes and macrophages, which release IL-1β, after the activation of pyroptosis, and in turn enhances NETs formation in various diseases by positive feedback [78,79,80,81,82] (Figure 2). This positive feedback may be activated in severe COVID-19 patients, accelerating NETs and non-canonical pyroptotic interleukin IL-1α production, and inducing respiratory decompensation, DIC, and abnormal immune responses [34].

Furthermore, high NETs levels in plasma correlate with an increase in IL-1α, IL-1β and IL-6, and adhesion molecules such as P-Selectin (P-SEL) [82] (Figure 2). Notably, IL-1α induces IL-6 expression, [83] which has emerged as a promising target for COVID-19 treatment [44]. However, both positive [84] and negative results [85] have been reported regarding the use IL-6 blockers to treat COVID-19.

One of the reasons for using IL-6 blockers (e.g., tocilizumab) is that IL-6 induces fibrinogen expression. After being activated by thrombin, fibrinogen converts to fibrin, a key mediator in blood coagulation and inflammatory response that could increase the procoagulant effect [86,87]. Also, fibrin and NETs interact to form a composite network within thrombi [88].

To conclude, further research on the role of monocytes and macrophages mediating NETosis is therefore expected to shed light on the pathophysiology of the COVID-19-associated inflammatory response [33,89].

## 3. Critically Ill COVID-19 Patients Show Clinical Manifestations of Sepsis

The molecular factors that trigger severe illness in individuals infected by SARS-CoV-2 are not entirely understood and the development of severe COVID-19 does not seem to be related to viral load solely [90]. Therefore, it is essential to identify the molecular events associated with clinical features to predict COVID-19 severity in order to develop therapeutic strategies able to mitigate disease severity in critically ill patients. Increased levels of fibrin degradation products measured as D-dimer [9], DIC [6] and higher sequential organ failure assessment (SOFA) scores are hallmarks of organ injury in sepsis and are closely associated with poor prognosis in severe COVID-19 patients [6,9,31]. Clinicians are attempting to understand the heterogeneity of this disease and its clinical features [5] beyond their interest in preventing this pandemic from spreading. Moreover, it would be of special interest to rapidly identify critically ill cases susceptible to life-threatening situations or deaths and avoid a fatal disease course.

Severe COVID-19 patients also share some common characteristics with sepsis [4], such as inflammation, high levels of systemic pro-inflammatory cytokines, immune dysregulation and microthrombosis [34]. This is probably due to the increase in Ang II levels caused by the SARS-CoV-2 and ACE2 interaction itself, and/or the interconnection between the inflammation produced by high levels of IL-6 and other pro-inflammatory cytokines and the oxidative stress identified in COVID-19 patients, contributing to tissue damage. In fact, the interconnection of mechanisms such as inflammation and oxidative stress seems to be extremely important in determining the severity of COVID-19. For example, IL-6 overexpression and oxidative stress lead to increased fibrinogen processing and fibrin levels which, in turn, interacts with NET’s facilitating coagulation phenotypes, contributing to the COVID-19-associated coagulopathy [88,91,92] and shock [4]. Some patients also suffer neutrophilia, which results in increasing NET production and correlates with severe COVID-19 phenotypes and poor outcomes [72,73,74,75,76,93].

Moreover, under physiological conditions, ACE2 plays a key role in the homeostatic control of vascular tone and hypertension, and in controlling the severity of acute lung failure [94,95,96]. Moreover, ACE2 is expressed in arterial and venous endothelial cells [97,98], where it exerts an anti-inflammatory protective effect. It is noteworthy that vasoconstriction and DIC are typical characteristics of septic patients [99] as we described previously. However, whether the coagulopathy observed in COVID-19 patients is a consequence of the direct vascular endothelium damage induced by SARS-CoV-2 infection or due to ACE2 blockade remains unknown [100], although Libby and Lüscher have presented COVID-19 as an endothelial disease by providing a unifying pathophysiological picture of the illness [28], in which both inflammation and oxidative stress are relevant players in endothelial function.

The interaction of SARS-CoV-2 with organs that exhibit the liver/lymph node-specific intercellular adhesion molecule-3-grabbing integrin (L-SIGN) is being investigated as an additional receptor for the virus [101] (Figure 2). Furthermore, how the virus spreads to other organs and its variation in virulence remain unknown.

COVID-19 patients who suffer sepsis present altered mental state, dyspnea, reduced urine output, faster heart rate, a weak pulse, and cold extremities [4]. The symptoms mentioned above are likely brought on by low blood pressure and a hypercoagulation state by the direct and indirect action of SARS-Cov-2. Also, acidosis, high lactate, or hyperbilirubinemia have also been observed in most patients, which are all features related with oxidative stress mechanisms [102,103] and antioxidant responses [104]. Severe COVID-19 patients with developed typical clinical manifestations of septic shock, such as serum lactate levels >2 mmoL L^−1^ and persistent hypotension despite fluid resuscitation, generally require vasopressors to maintain MAP ≥ 65 mm Hg [105].

Regarding the hypercoagulation associated with COVID-19, microthrombi in lungs, lower extremities, brain [33,106], liver, kidneys, and heart [107] have been reported. In particular, DIC phenotype is a hallmark of sepsis, which is mainly mediated by inflammatory cytokines [108]. Regarding DIC, high IL-6 levels can induce fibrin formation after fibrinogen proteolysis stimulation, which, in turn, mediates the inhibition of physiological anticoagulants [109]. Oxidative stress may produce fibrinogen oxidation and clot formation, in so doing contributing to coagulopathy in COVID-19 patients [110]. In addition, fibrin formation is one of the contributors to the hypoxemia observed in patients with apparently functional lungs. If left untreated, clots filter out additional clotting factors from the bloodstream, which increases the risk of bleeding and multi-organ failure in severe patients [111]. In fact, the cytokine storm leads to acute lung injury and death [112,113].

Nevertheless, characterizing the individual clinical responses to specific treatments in each patient remains a challenge, mainly because of the extensive heterogeneity of clinical manifestations in patients during disease progression. Elucidating individual clinical responses can offer better patient care, improve the chances of survival, and even reduce short-term and long-term post-COVID-19 sequelae or persistent COVID-19 symptoms. Nowadays, there is no doubt about the complexity of COVID-19 in terms of transmission to humans and difficulty to slow down its spread. Likewise, the multilevel therapies necessary for overcoming the disease are still being investigated and continuously evolving. Acting on viral replication (remdesivir has shown beneficial effects) [114], controlling the inflammatory cascade (cytokine blockade, among others) [4] and reducing thrombi formation (anticoagulant therapies) are among the most commonly used therapeutic approaches to treat COVID-19. However, from our point of view, antioxidant molecules/drugs could help to attenuate COVID-19 clinical severity by inactivating or counteracting the deleterious effects produced by oxidative stress in COVID-19. Finally, new therapies based on avoiding NETosis, blocking inflammation mediated by NLRP3 inflammasome, or blocking the pathway activated through L-SIGN, could also be feasible strategies to improve critical characteristics related to COVID-19 [101,115].

## 4. Antioxidant Therapies in Severe Cases of COVID-19

Recent studies postulate that oxidative stress is an essential factor that increases the severity of COVID-19 in some patients [60,116], especially associated with pulmonary dysfunction [117] and the cytokine storm or in viral sepsis derived from SARS-CoV-2 infection. In this regard, oxidative stress is related to sepsis, as we noted in the previous section.

Paruchner reviewed several promising studies exploring the therapeutic potential of antioxidants in cellular and animal models of sepsis [118]. Unfortunately, the results when antioxidants were evaluated in clinical trials performed in human beings, did not always show the same promising results as those found in animal models. For example, Manzanares et al. conducted a meta-analysis describing the effects of antioxidants in critically ill patients concluding that these therapies (mainly based in selenium supplementation) reduce mortality (>10% mortality in the control group) and the requirement of mechanical ventilation. In contrast, they did not find any reduction in length of stay in ICU [119]. However, other meta-analysis conducted by Allingstrup and Afsharidid did not find clear evidence in favor of selenium supplementation for septic patient outcomes such as the number of days on a ventilator, length of ICU stay, or length of hospital stay and contradicting results were obtained for 28-day mortality [120], as well as the incidence of new infections, or on length of stay in ICU or mechanical ventilation [121].

Although the use of antioxidant strategy has been proposed as adjuvant therapy in sepsis, there is no antioxidant therapy approved by regulatory agencies and organizations [118], although several clinical trials are currently active. This is also the case with COVID-19, in which many antioxidants such as vitamins A, C, D, melatonin, resveratrol, reduced glutathione, N-acetylcysteine, silymarin, quercetin, artemisinin, curcumin, Boswellia, and hesperidin are currently being evaluated in clinical trials. Likewise, other drugs with antioxidant effects such as statins, colchicine, or amiodarone, have been suggested as part of the arsenal to treat, or at least attenuate, COVID-19 symptoms and sequelae [122,123,124,125]. Therefore, lessons learned in previous trials using antioxidants in sepsis may serve to hypothesize the therapeutic possibilities of these agents in COVID-19.

The use of antioxidants as a complementary therapeutic strategy in COVID-19 has been proposed [60,116]. A possible strategy could be focused on regulating the nuclear erythroid-related factor 2 (Nrf2) and Nrf2/antioxidant related elements (ARE), the main transcription factor involved in stimulating the enzymatic antioxidant defense [126]. In this regard, the possible therapeutic potential of antioxidants with the ability to activate Nrf2 (such as resveratrol, sulforaphane, melatonin and vitamin D) [127,128,129,130] may then be considered for both sepsis and viral sepsis occurring in severe COVID-19 patients [131].

Vitamin D functions by regulating cell signaling mediated by Ca2+ and ROS, playing a central role in the phosphorous homeostasis [132]. Moreover, vitamin D was proposed as one of the critical controllers of systemic inflammation by its function, regulating oxidative stress and mitochondrial respiratory function [133]. Additionally, vitamin D has an anti-inflammatory effect by controlling the adaptive immune system [134]. Furthermore, vitamin D induces the expression of several molecules involved in the antioxidant defense system including CAT, GPx, GSR, and SOD, increases the levels of reduced glutathione, and suppresses the expression of NADPH oxidase, thereby contributing to reducing oxidative stress and cellular oxidation [133]. Finally, vitamin D is known to elicit a vasoprotective effect [135].

Interestingly, in a recent retrospective study performed in 780 older male cases of COVID-19, low levels of vitamin D were associated with increased odds of death [136], suggesting that vitamin D supplementation could reduce the severity of COVID-19 [137]. However, this is a matter of debate and further randomized clinical trials should be performed to demonstrate its role as a therapeutic agent for COVID-19. At present, 48 clinical trials are registered in ClinicalTrials.gov in which vitamin D is being evaluated as a therapy for COVID-19. Among these, there are ten clinical trials in very advanced stages (phase 3 or phase 4) and the final results are expected to be known soon (Table 1). Some of them seek to determine whether a vitamin D supplement can reduce the rates of hospitalization and death of COVID-19 patients, as well as whether the intake of vitamin D reduces the risk of infection with the SARS-CoV-2 virus (NCT04536298) and the severity and duration of the disease (NCT04483635, NCT04535791) in pediatric patients (NCT04502667) and in older patients (NCT04344041). Other trials are evaluating the relationship between the initial deficiency of vitamin D and the clinical phenotypes of COVID-19 patients (NCT04385940 and NCT04411446) or are evaluating the therapeutic efficacy of rapidly correcting vitamin D deficiency in adults to reduce the risk of SARS-CoV-2 infection and mitigate the morbidity and mortality associated with COVID-19 (NCT04386850).

It should be considered that besides the role of vitamin D counteracting the deleterious effects of oxidative stress, vitamin D also contributes to the appropriate function of T-cells [138,139], therefore contributing to immune response.

Among the anti-oxidant molecules mentioned above, melatonin-based therapy stands out among other treatments. Melatonin (N-acetyl-5-methoxy-tryptamine) is synthesized from the tryptophan amino acid in the pineal gland and is secreted into the cerebrospinal fluid [140]. The direct free radical scavenging activity of melatonin has been understood for almost 30 years [141]. Melatonin is a natural hormone with an extraordinary ability to reduce oxidative stress in both natural and pathological circumstances [142]. In addition to its antioxidant capacities against ROS (including (^•^OH), H_2_O_2_, singlet oxygen (^1^O_2_), and RNS (such as peroxynitrite (HNOO)), it has also been proposed that it plays a role in activating different anti-oxidant enzymes, via Nrf2, and inhibiting the pro-oxidant activity of some enzymes [143,144,145,146,147]. Moreover, melatonin has demonstrated its potential as an anti-inflammatory agent in acute and chronic inflammatory processes [148,149].

Melatonin has been evaluated in sepsis because of its antioxidant, anti-apoptotic, anti-ferroptotic anti-inflammatory, and immunomodulatory properties, and because it is the only anti-inflammatory molecule able to block the two main pathways of the innate immunity, NF-κB and NLRP3 inflammasome [150]. Moreover, melatonin has demonstrated its role in protecting cells against hypoxia and hemoglobin denaturation [151], preventing free iron release. The administration of exogenous melatonin in animal models before the development of severe phenotypes has demonstrated a decrease in the inflammatory response by reducing the release of pro-inflammatory cytokines, such as the pyroptosis-released IL-1β, and an increase in anti-inflammatory cytokine IL-4 levels in blood serum [152].

Up to six clinical trials have been identified in ClinicalTrials.gov using melatonin solely or in combination with vitamin C as a therapeutic strategy for sepsis, but no information about results of these trials’ has been posted. For example, a clinical trial (NCT03295162) assessing the efficacy of melatonin as an adjuvant in the treatment of oxidative stress in septic preterm infants receiving 10 mg of melatonin was finalized, but the results have not been yet published; something similar occurred in a trial designed to establish the therapeutic efficiency of melatonin in adult patients with severe sepsis and septic shock (NCT01858909). In other completed trials designed to evaluate the effect of melatonin dose escalation in healthy volunteers as a potential treatment for sepsis, results were also not presented (NCT01724424). There is currently another active trial in the recruiting stage (NCT03557229) consisting of a randomized controlled clinical trial of melatonin-based antioxidant therapy in critically ill patients with septic shock. Even though clinical trials yielded no information on the potential use of melatonin in sepsis, published results have demonstrated that the administration of 20 mg of melatonin for three days in newborns with sepsis improved the symptoms [153]. Recently, a relationship between the degree of the inflammatory and oxidative response in septic patients, the expression of the clock gene *bmal1*, and the levels of melatonin has been demonstrated [154].

Importantly, it was demonstrated that melatonin decreases D-dimer levels in stress-induced coagulopathy [155], particularly in sepsis and COVID-19. However, melatonin was not able to prevent the DIC in sepsis [156]. So, it should be considered that the administration of melatonin in COVID-19 does not prevent COVID-19-related thrombosis

Six clinical studies are currently being developed to evaluate melatonin’s safety and efficacy in patients with COVID-19 (ClinicalTrials.gov) (Table 2). Some clinical trials are focused on evaluating the anti-inflammatory and antioxidant actions of melatonin and its potential to reduce the severity of COVID-19 (NCT04474483/NCT04530539). Other clinical trials seek to evaluate the potential of melatonin to reduce the inflammatory cascade characteristic of SARS-CoV-2 through the inhibition of NLRP3 inflammasome (NCT04409522). This is because melatonin inhibits NLRP3-mediated inflammation as well as other inflammatory effects derived from the inflammasome, inducing a systemic anti-inflammatory response, especially after severe inflammation [157], so it is expected that the inhibition of NLRP3 using melatonin may lead to a reduction in systemic inflammation and oxidative stress, and in so doing reducing the severe phenotypes in COVID-19 patients. As promoted by the Hospital Universitario La Paz Research Institute, other clinical trials seek to evaluate the efficacy of melatonin as prophylaxis in healthcare workers exposed to the virus in their clinical practice (NCT04353128). Another active trial evaluates the effect of melatonin plus toremifene (selective estrogen receptor modulator) on the signs and symptoms of COVID-19 patients (NCT04531748). An additional clinical trial is analyzing the adjuvant therapeutic effects of melatonin and its agonist ramelteon (NCT04470297). Additionally, a clinical trial with an injectable formulation of melatonin for intravenous perfusion in ICU patients suffering from COVID-19 was designed to learn about the doses and efficacy of melatonin against COVID-19 [150].

## 5. Conclusions

The precise molecular mechanisms that mediate the pathological inflammation, vascular dysregulation and coagulopathy derived from SARS-CoV-2 infection are only beginning to emerge. However, it is crucial to identify the overall pathways involved in the different inflammatory response stages during infection and how they contribute to the final patient outcome.

Although the whole representative data characterizing the immune and inflammatory status in COVID-19 patients is not completely known, oxidative stress, systemic hyperinflammation, and coagulopathy contribute to COVID-19 severity. Mechanisms like pyroptosis and NETosis, contributed by oxidative stress and inflammation, seem to underlie COVID-19 features. In this scenario, NETosis and pyroptosis are both activated by oxidative stress [61,77,158]. Moreover, some authors postulate that the overwhelming production of ROS in COVID-19 results in increased oxidative stress, which contributes to local and systemic tissue damage and inflammation contributing to severe clinical phenotypes of COVID-19 [77] inducing a vicious cycle that establishes the progression of the disease to sepsis [4].

Clinically speaking, and even though the vast majority of critically ill COVID-19 patients develop sepsis, which has been postulated as the real cause of most deaths, the molecular mechanism by which SARS-CoV-2 mediates sepsis remains unclear. Therefore, understanding some of the molecular mechanisms described in this review and how these mechanisms are controlled or produce oxidative stress and hyperinflammation are of particular relevance to the effort to design potential therapeutic strategies for COVID-19 treatment. So, considering that oxidative stress is emerging as a critical element in the physiopathology of COVID-19, antioxidants may be feasible co-adjuvant therapeutic agents to attenuate COVID-19 severity.

Clinical trials using antioxidant therapies based on the use of vitamin D and melatonin are currently underway. Treatment with vitamin D appears promising because of its role as antioxidant, immunomodulatory, and regulator of vascular homeostasis, but more data are needed to corroborate its supposed efficacy in COVID-19. Of note, melatonin, through its anti-inflammatory, antioxidant, and immunomodulatory properties, is a promising candidate as a co-adjuvant treatment in COVID-19 [149,150]. However, as described in the previous section, we must wait for the published results of the ongoing clinical trials.

Furthermore, we need to be cautious, because many antioxidant-based therapies in humans have produced negative or inconclusive results or have demonstrated only minimal benefit [159]. This is probably because of the complexity of many diseases in which oxidative stress is not the only contributor to the physiopathology and/or because we do not know enough about the appropriate concentration or timing of treatment with the antioxidant drug, which may not be safe or bioavailable for a specific therapeutic effect in its appropriate tissue or molecular target [160]. Moreover, other aspects should also be considered so as to increase the success of clinical trials based on antioxidant therapies. In this regard, we propose the following strategies: (i) completely characterize the appropriate form of delivery, stability, and bioavailability of the antioxidant compound, (ii) perform clinical trials using cohorts with a large number of enrolled patients and/or, (iii) identify the characteristics of the subjects enrolled in clinical trials (e.g., lifestyle, feeding habits and vitamin supplement intake, baseline levels of antioxidants, smoking habits, morbidities, and treatments) in order to select appropriate subjects to be included in the clinical trial, (iv) identify the effective concentrations of antioxidants with enough potential to scavenge free radicals, complementing the enzymatic [160] and non-enzymatic defense system, and (v) characterize the genetic (and epigenetic and metabolomic) background of subjects by implementing personalized medicine in order to identify redox-sensitive signaling pathways altered not only in a specific disease, but also in individual subjects. These considerations are of particular importance in complex and heterogeneous diseases such as COVID-19, especially in those severe phenotypes progressing to sepsis in which antioxidant therapy could serve as an adjuvant therapy to mitigate the disease severity in critically ill cases.

## Figures and Tables

**Table 1:** More advanced active clinical trials using vitamin D as therapeutic agent for COVID-19.

Clinical Trial ID	Development Phase	Aim
NCT04536298	Phase 3	The work is carried out in patients recently diagnosed with Covid-19 and the goal lies in the intake of a nutritional supplement of vitamin D to evaluate an improvement in patients (reducing the rates of hospitalization and death). Likewise, this study states that the intake of vitamin D reduces the risk of infection with the SARS-CoV-2 virus.
NCT04385940	Phase 3	The objective of this three-week study in patients with Covid-19 is to determine the relationship between initial deficiency of vitamin D and the clinical characteristics of patients, with improvement of the clinical phenotype in patients with vitamin D supplement.
NCT04483635	Phase 3	This 16-week study aims to demonstrate that patients infected with SARS-CoV-2 who ingest a high-dose of vitamin D supplementation decrease their incidence of infection and the severity and duration of the disease.
NCT04535791	Phase 3	The aim of this study is to evaluate the efficacy of vitamin D supplementation against virus infection for Covid-19 healthcare workers.
NCT04411446	Phase 4	The objective is to determine whether an oral dose of vitamin D is able to prevent the occurrence of respiratory derangement and other adverse clinical events in Covid-19 hospitalized patients.
NCT04502667	Phase 3	This study tests the efficacy of vitamin D treatment in Covid-19 pediatric patients
NCT04344041	Phase 3	This study is examining whether high-dose vitamin D supplementation improves the prognosis of older patients diagnosed with COVID-19 compared to a standard dose of vitamin D.
NCT04386850	Phase 2–3	This clinical trial is evaluating the therapeutic efficacy of rapidly correcting vitamin D deficiency in adults to reduce the risk SARS-CoV-2 infection and the viral infection, and mitigate the morbidity and mortality associated with COVID-19.

**Table 2:** Active clinical trials using melatonin as therapeutic agent for COVID-19.

Clinical Trial Id.	Development Phase	Aim
NCT04474483	Phase 2	The aim of the clinical study is to determine the potential role of melatonin as an anti-inflammatory and antioxidant molecule, and its influence on disease severity and prevention of progression in SARS-CoV-2 patients.
NCT04409522	Not Applicable	The goal of this study is to measure the efficiency of melatonin as a treatment alongside the common antiviral drugs in patients with severe Covid-19.
NCT04531748	Phase 2	This clinical study is designed to evaluate the role of melatonin by itself and with another drug (Toremifene) in the signs and symptoms of Covid-19 patients, compared to a placebo, in order to identify the best treatment.
NCT04530539	Not applicable	The aim of this study is to evaluate the influence of vitamin C and melatonin on the signs, symptoms, and outcomes of Covid-19 patients.
NCT04470297	Phase 2	This study is designed to test the clinical effects of an agonist of melatonin, ramelteon 8mg, in Covid-19 hospitalized patients.
NCT04353128	Phase 2–Phase 3	The aim of this clinical study is to evaluate the role of melatonin in preventing infection in people exposed to SARS-CoV-2. Thus the study focuses on evaluating its effectiveness.
